# Electrochemiluminescence of Semiconductor Quantum Dots and Its Biosensing Applications: A Comprehensive Review

**DOI:** 10.3390/bios13070708

**Published:** 2023-07-05

**Authors:** Hui Sun, Ping Zhou, Bin Su

**Affiliations:** Key Laboratory of Excited-State Materials of Zhejiang Province, Institute of Analytical Chemistry, Department of Chemistry, Zhejiang University, Hangzhou 310058, China; 11937052@zju.edu.cn (H.S.); zhouping3241@163.com (P.Z.)

**Keywords:** electrochemiluminescence, quantum dots, mechanism, coreactant, biosensor, immunoassay, nucleic acid analysis, small molecules detection

## Abstract

Electrochemiluminescence (ECL) is the chemiluminescence triggered by electrochemical reactions. Due to the unique excitation mode and inherent low background, ECL has been a powerful analytical technique to be widely used in biosensing and imaging. As an emerging ECL luminophore, semiconductor quantum dots (QDs) have apparent advantages over traditional molecular luminophores in terms of luminescence efficiency and signal modulation ability. Therefore, the development of an efficient ECL system with QDs as luminophores is of great significance to improve the sensitivity and detection flux of ECL biosensors. In this review, we give a comprehensive summary of recent advances in ECL using semiconductor QDs as luminophores. The luminescence process and ECL mechanism of semiconductor QDs with various coreactants are discussed first. Specifically, the influence of surface defects on ECL performance of semiconductor QDs is emphasized and several typical ECL enhancement strategies are summarized. Then, the applications of semiconductor QDs in ECL biosensing are overviewed, including immunoassay, nucleic acid analysis and the detection of small molecules. Finally, the challenges and prospects of semiconductor QDs as ECL luminophores in biosensing are featured.

## 1. Introduction

Electrochemiluminescence (ECL) is the chemiluminescence triggered by electrochemical reactions [1,2]. The combination of electrochemistry and chemiluminescence brings ECL unique advantages [3]. The absence of the excitation light source allows ECL to avoid the interference of stray light and background fluorescence that is always present in photoluminescence (PL), thus achieving a high signal-to-noise ratio. The unique electrochemical excitation method makes ECL spatiotemporally controllable and recyclable, thereby it is superior to chemiluminescence (CL) in stability and durability. Thanks to the above advantages, ECL has manifested itself as a powerful analytical technique in the fields of immunoassay [4,5], molecular diagnostics [6,7] and biological imaging [8,9,10].

Tris(2,2′-bipyridyl)ruthenium(II) ([Ru(bpy)_3_]^2+^) and luminol are typical ECL luminophores in plenty of research works [6]. Particularly, the [Ru(bpy)_3_]^2+^/tripropylamine (TPrA) system has been successfully commercialized in clinical immunodiagnostics due to the so-called low-oxidation-potential ECL pathway, enabling sensitive detection of hundreds of biomarkers. Nowadays, immunoassay based on the [Ru(bpy)_3_]^2+^/TPrA system still remains one of the most significant applications of ECL. However, it is undeniable that the system still has drawbacks. For example, the low photoluminescence quantum yield (PLQY) of [Ru(bpy)_3_]^2+^ (~4%) limits the improvement of ECL efficiency, and the difficulty of modulating the wavelength and broad emission spectrum (~200 nm) also leads to low throughput [11]. In order to address these issues, a lot of research work has been carried out to develop novel highly efficient ECL luminophores [12,13].

Quantum dots (QDs) are colloidal nanocrystals with geometric dimensions smaller than the exciton Bohr radius of corresponding bulk materials [14,15]. Due to the quantum confinement effect, QDs possess excellent optical properties, such as a broad absorption spectrum, narrow emission spectrum and continuously tunable luminescence wavelength with size and composition, showing great prospects in ECL biosensing [16,17]. Since Bard and coworkers first observed the ECL phenomenon of Si QDs in 2002 [18], ECL studies of QDs with various structures and compositions have been conducted, and ECL generation from CdSe [19], CdSe/ZnSe [20], CdTe [21] and Ge QDs [22] has been reported in succession. Since then, ECL luminophores have been expanded from organic/inorganic molecules to nanomaterials. In the past two decades, the fast-growing synthesis chemistry and spectral characterization techniques of QDs have facilitated the in-depth investigation of their ECL properties [16,23]. The ECL mechanism of QDs with several highly effective coreactants and its applications in biosensing have been widely explored [12,13].

Compared with traditional organic/inorganic ECL luminophores, the excellent optical properties of QDs give them more advantages in the construction of ECL biosensors. In general, [Ru(bpy)_3_]^2+^ and its derivatives emit light only in the orange-red region of the visible spectra [6], and it is difficult to perform multiplex immunoassay relying on the tunable spectra of ruthenium(II) complexes. Although the spectra of iridium (III) complexes can be modulated by changing the ligand structure, their spectral linewidth is typically big, thus limiting the improvement of detection flux [24,25]. In order to overcome these challenges, Su et al. developed a potential-resolved multicolor ECL biosensor for multiplex immunoassay in a single sample [4]. Several ruthenium (II) and iridium (III) complexes with distinguishable ECL emission wavelengths and potentials were synthesized and used for the simultaneous recognition of three antigens in a single measurement. But this strategy still has some limitations. There are only two luminophores being spectrum-resolved in the wavelength range of 400–900 nm.

By contrast, the emission wavelength of QDs can be continuously modulated with the variation of size and composition, showing the potential of QDs in ECL multiplex immunoassay. Moreover, the solubility and surface ligands of QDs can be easily regulated, providing QDs good biocompatibility. Zou et al. first realized the spectrum-resolved triplex-color ECL multiplex immunoassay using multicolor QDs as ECL emitters [26]. Three QDs with different size and composition were directly conjugated with corresponding antibodies by amidation and their ECL signals were resolved in the range of 500–900 nm. Su and Peng et al. synthesized green-, yellow-, and red-emitting CdSe/CdS/ZnS core/shell/shell QDs with bright and stable ECL emissions [27]. Thanks to the outstanding band-edge ECL performance of CdSe/CdS/ZnS QDs, ECL signals of three QDs could be distinguished in the range of 450–750 nm, showing great promise in ECL multiplex biosensing.

However, due to the complexity of structures, there are still some ambiguous problems and obstacles in the study of ECL mechanism and applications of QDs. QDs consist of a core and surface ligands [28]. With the size of the core gradually decreasing to below the exciton Bohr radius, the quantum confinement effect becomes particularly significant, and simultaneously the luminescence wavelength shifts hypochromatically [23]. At the same time, the decrease in size will lead to a sharp increase in the proportion of surface atoms in the total number of atoms, and then the surface defects formed by surface atoms containing unbonded electrons will strongly affect the luminescence process of quantum dots, resulting in the quenching of band-edge excited states and reduction in PLQY. The most effective way of passivating surface defects is to epitaxially grow a shell with a larger band gap or to introduce surface ligands. However, since the excited state generated in the ECL process originates from the charge transfer between QDs and electrodes or coreactants, the introduction of large-band gap shells and insulating organic ligands will inevitably lead to a decrease in the charge transfer rate and thus a decrease in ECL efficiency [29,30]. Therefore, there has to be a trade-off between the improvement of optical properties and the acceleration of charge transfer, which is crucial to improve the ECL efficiency of QDs for better applications in highly sensitive biosensing.

Though QDs are promising ECL emitters, some problems are yet to be resolved considering the complexity of their structures, which limits the further development of their applications in biosensing. Herein, we shall give a detailed summary of recent advances in ECL using semiconductor QDs as luminophores, and then their mechanisms of ECL generation with various coreactants will be introduced. In the following sections, on the basis of understanding the ECL mechanism of QDs, we are about to review the recent applications of QDs as ECL luminophores in the fields of immunodiagnosis, nucleic acid analysis, and the detection of small molecules and ions. Finally, the challenges and prospects of QDs as ECL luminophores in biosensing will be discussed.

## 2. ECL System of QDs

For the ECL process of QDs, the formation of excited states relies on the sequential injection of electrons and holes (or vice versa) into the ground states of QDs. The donors of electrons and holes can be both electrodes or electrodes and coreactants, respectively. In this section, we review the research progress on ECL systems using QDs as luminophores and corresponding reaction pathways, including annihilation and coreactant ECL mechanisms.

### 2.1. QDs as ECL Luminophores

Since Bard’s group reported a series of pioneering works on the ECL of metal chalcogenide QDs, QDs with different structures and compositions as ECL emitters have been extensively studied [18,19,20,21,22,31]. Inorganic semiconductor QDs were the earliest studied and most widely used materials. With mature synthetic strategies and alternative shell materials with a tunable band gap, it is easy to prepare inorganic semiconductor QDs with excellent optical properties and continuously adjustable emission wavelengths [23]. After that, ECL generation from a series of novel nanomaterials such as carbon QDs [32], graphene QDs [33], halide perovskite QDs [34], SnO_2_ QDs [35] and MoS_2_ QDs [36] was reported, which greatly enriched the types of QDs used in ECL studies. However, semiconductor QDs are still the most widely used QDs in ECL, so this review will focus on the ECL properties and applications of semiconductor QDs. The QDs mentioned below refer to semiconductor QDs unless otherwise specified.

The excellent optical properties of QDs are the basis of highly efficient ECL. Therefore, in the past two decades, the development of ECL using inorganic semiconductor QDs as emitters is closely related to the optimization of synthesis strategies. Due to the large surface-to-volume ratio and high proportion of surface atoms, it is easy to introduce lattice defects and surface defects in the synthesis process of QDs, thus resulting in the generation of additional defect-state energy levels. Since the band gap of defect states is generally smaller than that of the band-edge state, the luminescence arising from the defect state is sensitive to the environment and has poor controllability. In addition, the emission spectrum from the defect state is generally red-shifted and broadened significantly compared with that from the band-edge state, and the luminescence efficiency decreases accordingly [37].

In the early research works of ECL generation by inorganic semiconductor QDs, QDs are synthesized in organic phase using dodecylamine or trioctylphosphine (TOP) as surface ligands, which therefore have a good solubility in organic solvents. Accordingly, early studies on ECL properties of QDs were mostly carried out in organic solvents such as acetonitrile, methylene chloride and *N*,*N*′-dimethylformamide (DMF) [18,19,20,21,22]. However, limited by the poor synthesis methods, the early QDs used in ECL research usually had many defects, resulting in low ECL performance and ambiguous ECL mechanism. There are also a lot of controversies on whether the ECL of QDs originated from the defect state or the band-edge state.

**Figure 1:**
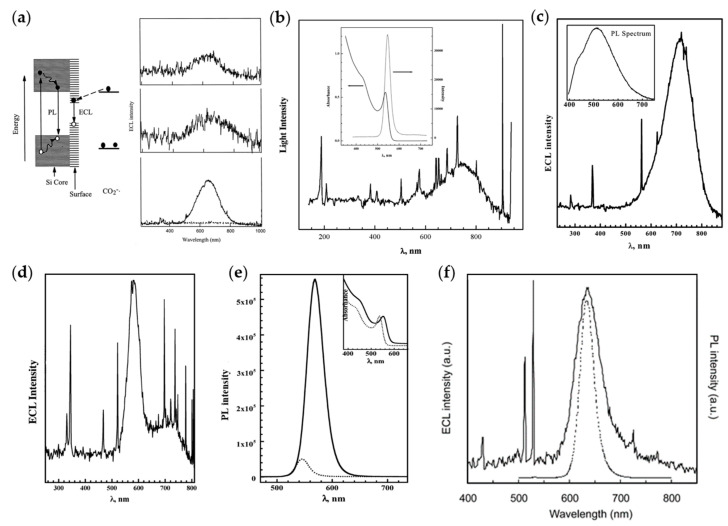
Early research works on ECL of QDs in organic phase. (**a**) Left: Schematic mechanisms for ECL and PL of Si clusters; Right: ECL spectra of Si nanocrystals in annihilation pathway (top) and coreactant pathway with oxalate (middle) or persulfate (bottom) as coreactants. Reprinted with permission from ref. [18]. Copyright 2002 The American Association for the Advancement of Science. (**b**) ECL and PL spectra of CdSe nanocrystals. Reprinted with permission from ref. [19]. Copyright 2002 American Chemical Society. (**c**) ECL and PL spectra of Ge nanocrystals. Reprinted with permission from ref. [22]. Copyright 2004 American Chemical Society. (**d**,**e**) ECL (**d**) and PL (**e**) spectra of CdSe/ZnSe nanocrystals. Reprinted with permission from ref. [20]. Copyright 2003 American Chemical Society. (**f**) ECL (solid line) and PL (dotted line) spectra of CdTe nanocrystals. Reprinted with permission from ref. [21]. Copyright 2004 American Chemical Society.

In the first work using Si QDs as ECL emitters reported by Bard’s group in 2002 [18], the ECL spectrum was centered at 640 nm, showing a significant red shift from the PL peak (~420 nm). This phenomenon led to a conjecture that PL originates from band-edge states while ECL originates from surface states (Figure 1a). Subsequently, Bard’s group further confirmed this conjecture by exploring the ECL performance of CdSe QDs and Ge QDs. The ECL spectra of QDs were found to be red-shifted by ~200 nm in comparison with the PL spectra, implying that ECL emission of QDs predominantly arises from surface states, in agreement with previous studies (Figure 1b,c) [19,22]. However, a series of subsequent studies overturned this conclusion. Bard’s group believed that surface states are quenchers of luminescence and the passivation of surface defects is the key to the preparation of high-quality QDs. Thus, a shell of ZnSe with a wider band-gap was grown on the surface of the CdSe core in order to passivate the surface defects. The ECL spectrum of CdSe/ZnSe QDs displayed two peaks: a sharp one coincident with the PL spectrum and another broad one with a red shift of ~200 nm compared to the PL spectrum, arising from surface states and band-edge states, respectively. And the passivation of surface states favored the generation of band-edge emission (Figure 1d,e) [20]. Later, the ECL spectrum of CdTe QDs was found to locate at the same position as the PL spectrum, but slightly wider than that of the PL spectrum, indicative of the negligible involvement of surface states in ECL process of CdTe QDs (Figure 1f) [21]. Based on the above research works, it can be concluded that ECL emission of semiconductor QDs can come from both surface defect states and band-edge states, and the proportion of them in the luminescence process may depend on the passivation degree of surface defects.

As summarized above, the early explorations of ECL properties of QDs were always carried out in organic solvents. However, since biosensing usually needs to be proceeded in aqueous solutions, it is of great necessity to develop ECL systems with QDs as emitters in aqueous solutions for realizing the practical application of QDs in biosensing. Weller’s group reported for the first time the band-edge ECL of CdSe and CdSe/CdS QDs in aqueous solutions [38]. The n-doped QDs and SO_4_^•−^ are generated by electrochemical reductions when a negative potential is applied to the electrode coated with QDs and immersed in aqueous solutions containing K_2_S_2_O_8_ as coreactants; subsequently, the formed SO_4_^•−^ injects a hole into the valence band of n-doped QDs; eventually, the injected holes recombine with electrons from the conduction band and emit light. The ECL spectra of the above QDs coincide with corresponding PL spectra, indicating that the ECL emission originates from band-edge states. Since then, the ECL system of QDs has been extended from the anhydrous phase to the aqueous phase. Ju et al. reported the first QD-based ECL sensor in aqueous solutions [39]. CdSe QDs were synthesized directly in aqueous solutions by an ultrasonic procedure, and then a CdSe QDs film modified carbon paste electrode was observed to produce two strong ECL emissions at −1.20 V (ECL-1) and −1.50 V (ECL-2), respectively, in phosphate buffered saline (PBS). Compared with ECL-2, ECL-1 showed a higher sensitivity for the detection of oxidizing coreactants (e.g., H_2_O_2_). Later, Zhu et al. prepared a series of zinc-based nanocrystals such as ZnSe, ZnO/ZnS and ZnO/ZnSe in aqueous solutions through a newly developed ultrasound-assisted synthesis procedure, which promoted the development of the analytical application of QDs as ECL emitters in aqueous solutions [40].

**Figure 2:**
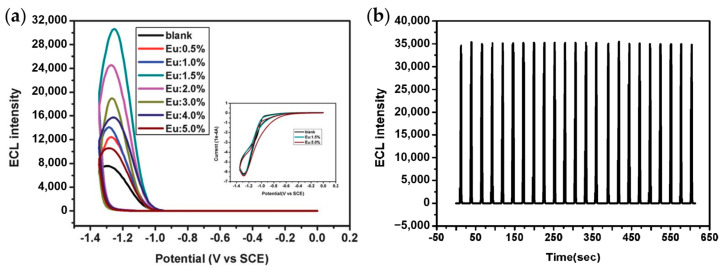
(**a**) ECL curves showing the dependence of ECL intensity on the doping level of Eu^3+^ in CdS QDs. (**b**) ECL behaviors of CdS:Eu QDs (1.5%) under continuous potential scan for 23 cycles from 0 to −1.35 V. Reprinted with permission from ref. [44]. Copyright 2012 Royal Society of Chemistry.

QDs synthesized directly in aqueous solutions tend to have poor optical properties and low ECL efficiency due to the low crystallization temperature. In order to improve the ECL properties of QDs dispersed in aqueous solutions, researchers explored a series of ECL enhancement strategies, including metal ion doping, surface passivation with bidentate ligands, construction of core-shell structures and complexation with other structures such as carbon nanotubes and graphene oxides [41,42,43]. Xu et al. studied the ECL properties of ZnS QDs doped with Mn^2+^ in aqueous solutions using H_2_O_2_ as a coreactant for the first time [42]. Although the ECL intensity of Mn^2+^-doped ZnS QDs was not significantly enhanced, the introduction of Mn^2+^ surface states caused the onset potential of ECL generation to shift considerably, thus avoiding the decomposition of ECL intermediates at a negative potential. Later, it was found that Eu^3+^ was more effective than Mn^2+^ as a dopant in improving the ECL properties of QDs [44]. As shown in Figure 2, the doping of Eu^3+^ causes a four-fold enhancement in ECL intensity and more stable cathodic signals compared with pure CdS QDs.

**Figure 3:**
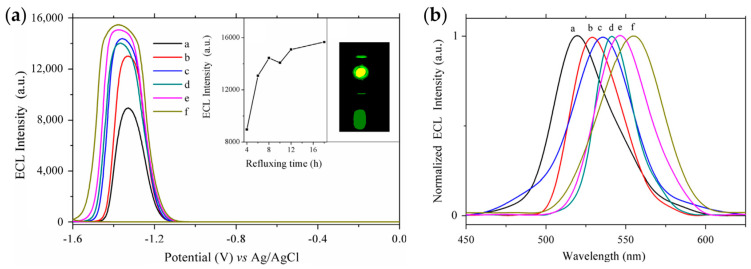
The dependence of ECL behaviors (**a**) and normalized ECL spectra (**b**) of dual-stabilizers-capped CdSe QDs on the refluxing time of (a) 4, (b) 6, (c) 8, (d) 10, (e) 12 and (f) 18 h. Reprinted with permission from ref. [43]. Copyright 2014 Elsevier.

The introduction of bidentate ligands is also an effective strategy for passivating surface defects and enhancing ECL. Zou et al. synthesized a series of Cd-based chalcogenide QDs protected by dual stabilizers in aqueous solutions, showing the importance of surface passivation for enhancing ECL [43,45]. CdTe QDs were synthesized in aqueous medium using sodium hexametaphosphate (HMP) and mercaptopropionic acid (MPA) as dual stabilizers, leading to enhanced ECL intensity and stability [45]. Subsequently, a series of CdSe QDs with emission wavelengths ranging from 523 nm to 556 nm were obtained by adjusting the reflux time during the synthesis process [43]. Compared with conventional CdSe QDs prepared in aqueous solutions, the dual-stabilizers-capped CdSe QDs displayed not only improved PL performance with a high PLQY (up to 29%) but also enhanced ECL emissions. And the ECL spectrum basically coincided with the PL spectrum, showing the importance of surface passivation (Figure 3).

**Figure 4:**
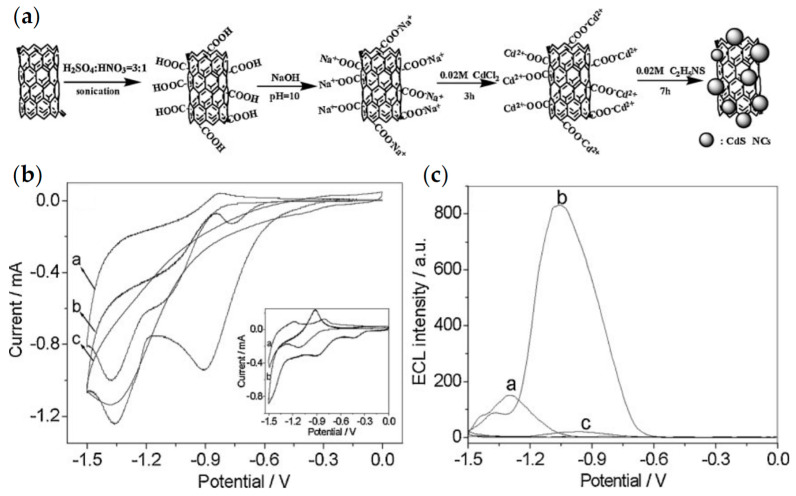
(**a**) Procedure of the preparation of CdS QDs on the modified multi-walled carbon nanotubes (MWCNTs); (**b**) CVs of (a) CdS QDs modified paraffin-impregnated graphite electrode (CdS-PIGE), (b) MWCNT-CdS-PIGE and (c) MWCNT-PIGE in 0.1 M PBS (pH 7.4) containing 1 mM H_2_O_2_. (**c**) ECL behaviors of (a) CdS-PIGE and (b) MWCNT-CdS-PIGE in 0.1 M PBS (pH 7.4) containing 1 mM H_2_O_2_ and (c) MWCNT-CdS-PIGE in 0.1 M PBS (pH 7.4) without H_2_O_2_. Reprinted with permission from ref. [46]. Copyright 2009 WILEY-VCH Verbg GmbH & Co. KGaA, Weinheim.

To improve the detection performance of QD-based ECL biosensors, researchers combined QDs with other nano-functional materials such as carbon nanotubes [41,46] or graphene oxides [47,48]. Xu et al. proposed the first signal-on ECL enzyme biosensor based on CdS QDs formed in situ on the surface of multi-walled carbon nanotubes (MWCNTs) [46]. As shown in Figure 4, the nanocomposites exhibited 5.3-fold enhanced ECL emissions and positively shifted onset potential of ~400 mV compared to pure CdS QDs. A less negative onset potential reduced the degradation of coreactants and thus improved the sensitivity of the CdS QD-based ECL biosensor. After that, Ju et al. developed a signal amplification system for CdTe QD-based ECL by using electrochemically reduced graphene oxide (ERGO) to construct a nanocomposite biosensing platform [48]. The ECL emission of CdTe QDs could be quenched by graphene oxide (GO) due to the structural defects but significantly enhanced by ERGO, which was mainly attributed to the strong adsorption of dissolved O_2_ by ERGO and the accelerated charge transfer process.

**Figure 5:**
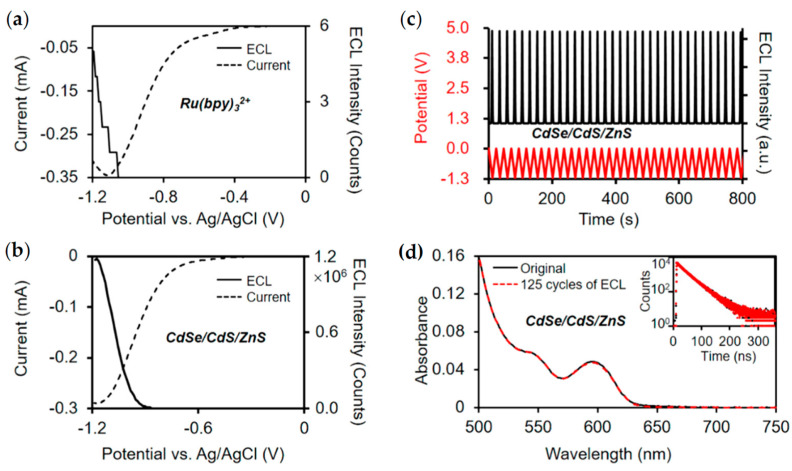
ECL performance of CdSe/CdS/ZnS QDs. (**a**,**b**) Current and ECL intensity curves of CdSe/CdSe/ZnS QDs and Ru(bpy)_3_^2+^ under identical conditions. (**c**) Stability of ECL generation over multiple cycles of potential sweeping between 0 and −1.2 V. The red curve represents the variation of externally applied potential, and the black curve shows the recorded ECL intensity. (**d**) Steady-state absorption and transient PL (inset) spectra of QDs before and after 125 cycles of potential scan between 0 and −1.2 V. The black curves and red curves represent the original data and the data after 125 cycles of ECL test, respectively. Reprinted with permission from ref. [27]. Copyright 2020 American Chemical Society.

Although ECL performance of QDs synthesized directly in aqueous solutions can be improved through various strategies, their ECL emission still mostly originates from surface defect states and it is still difficult to achieve efficient band-edge emission of QDs. Su and Peng et al. developed a novel QDs synthesis strategy in which CdSe/CdS/ZnS core/shell/shell QDs were synthesized in the organic phase and then transferred to the aqueous phase through surface ligand exchange [27]. As shown in Figure 5, the ECL intensity of CdSe/CdS/ZnS QDs is found to be 4.7 × 10^5^ times higher than that of Ru(bpy)_3_^2+^ under identical conditions, and also remains stable and reproducible during multiple cycles of potential scans between 0 and −1.2 V. A high synthesis temperature can give good crystallinity and excellent optical properties. Moreover, the core/shell/shell structure passivates the surface defects, thus generating efficient band-edge ECL emission. Finally, surface ligand exchange from carboxylate to mercaptopropionic acid without changing the core structure guarantees a good dispersibility in aqueous solutions, showing the potential for biosensing applications. Current research works on ECL properties of inorganic semiconductor QDs indicate that they are promising luminophores, but more efforts need to be devoted to the improvement of the band-edge ECL properties of QDs.

In addition, the acceleration of charge transfer between QDs and coreactants is also of vital importance for the generation of highly efficient ECL. Several research works on charge transfer within QDs–small molecules complexes have proved that both inorganic shells and surface ligands significantly hinder the process of charge transfer, and the effect of surface ligands on charge transfer is much greater than that of inorganic shells [29,30]. Therefore, shortening the surface ligands is an effective strategy to accelerate charge transfer and enhance the ECL efficiency of QDs. Luo et al. proposed an electrochemical gelation method for assembling metal chalcogenide QDs into a three-dimensional gel in which each QD was accessible to the ambient [49]. In addition, Cai et al. developed a novel water-induced gelation strategy in which CdSe QDs could be self-assembled into an all-inorganic aerogel [50]. The removal of surface ligands and direct connection of the core enabled charge transport within the aerogel network, and the resulting CdSe QD aerogel exhibited a 126-fold enhanced ECL intensity compared with CdSe QDs.

### 2.2. ECL Pathways of QDs

The ECL pathways of QDs include the annihilation and coreactant routes according to the different ways of applying potential. Since annihilation ECL requires the solvent to have a wide potential window, it is generally proceeded in organic solvents and mainly used in early research works. Subsequently, the introduction of coreactants overcomes the limitation of potential windows and expands the ECL system of QDs from the organic phase to the aqueous phase. Coreactant-containing ECL has gradually become the most important way for ECL generation by QDs at present. In this section, we discuss the mechanism of ECL generation using QDs as emitters under different reaction pathways and summarize the most commonly used coreactants in ECL generation by QDs.

#### 2.2.1. Annihilation ECL

The annihilation ECL generates excited states (QD*) by charge transfer between QD^+•^ produced by electrochemical oxidation and QD^−•^ by electrochemical reduction. Typical reaction steps include:

(1) $$\mathrm{QD}+e^{-}\to {\mathrm{QD}}^{-•}$$

(2) $$\mathrm{QD}-e^{-}\to {\mathrm{QD}}^{+•}$$

(3) $${\mathrm{QD}}^{-•}+{\mathrm{QD}}^{+•}\to {\mathrm{QD}}^{*}+\mathrm{QD}$$

(4) $${\mathrm{QD}}^{*}\to \mathrm{QD}+h\upsilon$$

Therefore, it is necessary to apply positive and negative potential pulses successively on the same electrode or positive and negative potential, respectively, on two electrodes to ensure that QD^+•^ and QD^−•^ exist simultaneously and an effective electron transfer process between them occurs [51,52]. The requirement for a wide potential window dictates that annihilation ECL can only be performed in organic solvents such as acetonitrile, methylene chloride and DMF [18,20,22], which greatly limits the development of analytical applications of QDs ECL in aqueous solutions.

#### 2.2.2. Oxidative-Reductive ECL

In the presence of coreactants, ECL of QDs can be generated by applying unidirectional potential on the electrode, which overcomes the limited potential window and the poor stability of anionic and cationic intermediates, improving the ECL performance of QDs significantly [12]. Depending on whether the applied potential is positive or negative, coreactant-containing ECL can be divided into two types: oxidative-reductive ECL and reductive-oxidative ECL. In the process of oxidative-reductive ECL, QDs and coreactants are oxidized to generate QD^+•^ and reductive radicals, respectively; then the excited states are formed after injecting electrons into the conduction band of QD^+•^ from the reductive radicals; eventually the recombination of electrons and holes results in the emission of light. The oxidative-reductive route is the most common pathway for the generation of ECL by inorganic complexes. The anodic ECL generated by QDs is analogous to that of inorganic complexes such as [Ru(bpy)_3_]^2+^. For the ECL generation of QDs, although the ECL efficiency of the oxidative-reductive pathway is usually lower than that of the reductive-oxidative one, the positive potential required for the generation of oxidative-reductive ECL can avoid the reductive destruction of transparent oxide electrodes (such as the most commonly used indium tin oxide and fluoride-doped tin oxide electrodes), which may be helpful for the enhancement of the stability of ECL signals.

The coreactants of oxidative-reductive ECL reported in the literature include oxalate (C_2_O_4_^2−^) [18], amines such as 2-(dibutylamino)ethanol (DBAE) and tripropylamine (TPrA) [53,54,55,56], sulfites (SO_3_^2−^) [57] and indium tin oxide (ITO) [47,58,59]. The detailed reaction steps and experimental conditions are summarized in Table 1.

#### 2.2.3. Reductive-Oxidative ECL

Reductive-oxidative ECL is more widely used in mechanism studies and biosensing applications than oxidative-reductive ECL because of the relatively high efficiency. In the process of reductive-oxidative ECL, both QDs and coreactants are reduced to form anionic intermediates, which then undergo charge transfer to generate excited states and emit light. In 2002, Bard’s group observed the generation of ECL in the negative potential region upon adding excess S_2_O_8_^2−^ to the solution, which was the earliest report on coreactant-containing ECL of QDs [18]. After that, intense ECL emission of CdTe QDs was observed at −1.85 V in CH_2_Cl_2_ containing 0.1 M tetra-*n*-butylammonium hexafluorophosphate (TBAPF_6_) as the supporting electrolyte, with ECL spectrum almost identical to PL [21]. Based on the research results of Ushida that CH_2_Cl^•^ produced by CH_2_Cl_2_ under irradiation plays a role as an electron acceptor to oxidize aromatic hydrocarbons [60], Bard et al. proposed that CH_2_Cl^•^ can act as the coreactant in ECL generation by CdTe QDs.

Later, reductive-oxidative ECL of QDs with H_2_O_2_ as a coreactant was conducted in aqueous solutions, which can be used for H_2_O_2_ biosensing [39,41,61]. In the ECL system of QDs with H_2_O_2_ as coreactants, HO^•^ as the oxidizing intermediate can inject holes into the valence band of QD^−•^ to form the excited states. Therefore, molecules that generate HO^•^ through electrochemical reduction at an appropriate potential can be used as coreactants for ECL generation of QDs, such as dissolved oxygen that is abundant in aqueous solutions [39]. The reaction steps and experimental conditions of reductive-oxidative ECL systems are summarized in Table 2.

## 3. Applications of QDs in ECL Biosensing

As discussed above, QDs, as novel ECL luminophores with high efficiency, stability and tunable emission wavelength, hold great promise for ECL biosensing to improve analytical sensitivity and throughput. Extensive research works on the application of QDs in ECL biosensing have been carried out almost at the same time as the studies on ECL mechanisms [62,63,64,65]. In this section, based on various sensing strategies, we give a detailed summary of the applications of QD-based ECL in immunoassay, nucleic acid analysis, and the detection of small molecules and ions.

### 3.1. Immunoassay

#### 3.1.1. Immunoassay Based on Antigen-Antibody Recognition

Specific antigen-antibody recognition is the basis of immunoassay. Due to the high affinity and specificity of antigen-antibody interaction proved by various characterization techniques [66,67], it has been widely used in the construction of ECL-based immunosensors with QDs as luminophores. According to the difference of immune structures, immunosensors based on antigen-antibody recognition can be divided into “antibody-antigen” and “antibody-antigen-antibody” formats, and the latter one is generally referred to as sandwich structure.

**Figure 6:**
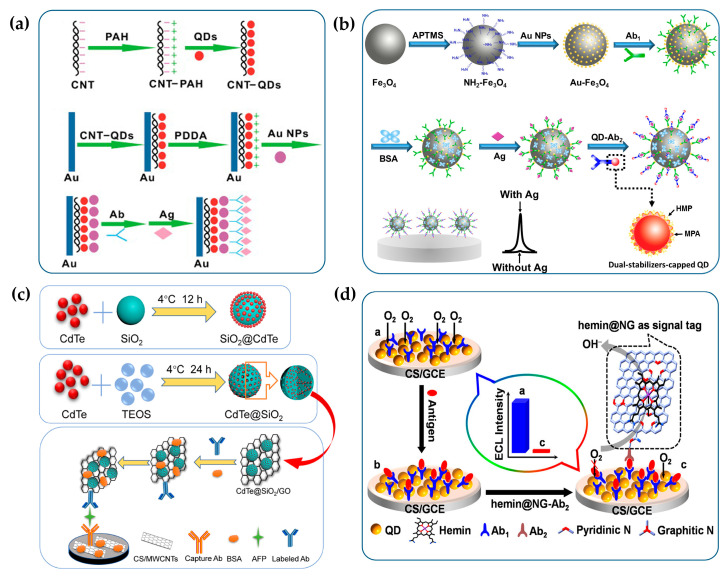
The fabrication procedures of ECL immunosensors based on antigen-antibody recognition. (**a**) The ECL immunosensor based on the steric hindrance effect. Reprinted with permission from ref. [69]. Copyright 2009 Elsevier. (**b**) The “signal-on” ECL immunosensor constructed on the magnetic solid substrate. Reprinted with permission from ref. [73]. Copyright 2017 Elsevier. (**c**) The “signal-on” ECL immunosensor based on dual signal amplification strategy by forming hybrid QDs materials with other nanoparticles. Reprinted with permission from ref. [74]. Copyright 2019 Elsevier. (**d**) The “signal-off” ECL immunosensor based on competitive consumption of coreactants. Reprinted with permission from ref. [75]. Copyright 2013 American Chemical Society.

Due to the simplified structures and no requirements for complex antibody labeling techniques, “antibody-antigen” type immunosensors were first proposed and widely used for sensitive detection of lipoprotein, human prealbumin, human IgG and carcinogenic antigens [62,68,69,70]. The construction procedures of “antibody-antigen” immunosensors are depicted in Figure 6a. QDs are first bound to the electrode through surface ligand functionalization and then antibodies are covalently conjugated to the electrode coated with QDs, followed by the block of non-specific binding sites with Bovine Serum Albumin (BSA) to reduce background interference. In the absence of antigens, QDs exhibit intense ECL because of the effective charge transfer. In the presence of target antigens, the immunocomplex formed by the specific recognition between antigen and antibody increases the steric hindrance and inhibits the charge transfer from coreactant radicals to QDs, resulting in a decrease in ECL intensity [69]. In addition, a variety of nanomaterials such as TiO_2_ nanotubes and metal-organic frameworks (MOFs) were used to form composites with QDs to accelerate the electron transfer between QDs and electrodes, further improving the sensitivity of detection [71,72].

With the rapid development of antibody-labeling techniques, immunosensors with sandwich structures have gradually become the mainstream of QD-based ECL immunosensors. They can be further divided into “signal-on” and “signal-off” immunosensors according to the variation of ECL intensity with the concentration of targets. Among them, the former with a sandwich structure is the most classic ECL biosensing strategy, which has been successfully commercialized in the field of immunodiagnosis using [Ru(bpy)_3_]^2+^ as ECL labels. In view of the significantly superior optical properties of QDs over [Ru(bpy)_3_]^2+^, a variety of sandwich-structured “signal-on” immunosensors with QDs as labels were constructed to improve the analytical performance of ECL immunoassay [76,77,78,79,80,81,82]. In the construction of “signal-on” immunosensors, primary antibodies are typically bound to the solid substrate to capture antigens and QDs are used as signal labels for the conjugation with secondary antibodies. In the presence of antigens, secondary antibodies labelled with QDs are captured to the solid substrates through the formation of immunocomplexes and then the concentration of antigens can be correlated with the ECL intensity of QDs to realize the quantitative detection of antigens. It should be noted that the solid substrates employed to fix primary antibodies can be either electrodes or magnetic beads. Due to the high surface area to volume ratio, the ease of bioconjugation and the convenience of magnetic separation, magnetic materials are the ideal solid carriers for the construction of ECL immunosensors. For example, as shown in Figure 6b, the sandwich-structured ECL immunosensor with CdTe QDs as labels and magnetic Fe_3_O_4_-Au nanocomposites as solid substrates is fabricated for the detection of carcinoembryonic antigens (CEA), achieving a limit of detection at 1 pg/mL (calculated by the slope of the linear regression equation: Δ*I* = 16.61*c* + 19.06) [73].

In addition, due to the extremely low concentration of antigens in biological samples and the limited number of QDs labelled on secondary antibodies, various signal amplification strategies have been developed to improve the detection sensitivity of trace antigens [74,80]. As shown in Figure 6c, a novel immunosensing strategy for α-fetoprotein (AFP) is proposed to achieve ECL signal amplification by enriching QDs with SiO_2_ nanoparticles. Specifically, MWCNTs are first modified to the glassy carbon electrodes (GCE) to bind with the primary antibody and then CdTe QDs-functionalized SiO_2_ nanoparticles are combined with GO to conjugate the secondary antibody. MWCNTs can accelerate electron transfer between CdTe QDs and electrodes. The combination of CdTe QDs-functionalized SiO_2_ nanoparticles and GO can not only increase the ECL intensity of a single label but also provide sufficient binding sites for the conjugation of secondary antibodies. Dual signal amplification strategies significantly improve the sensitivity of AFP immunoassay. In addition, QDs can be enriched by encapsulating them in porous nanostructures, thereby amplifying ECL signals and improving detection sensitivity. For example, Liu et al. combined SnS_2_ QDs and MOFs into the construction of ECL immunosensors to achieve synergistic amplification of ECL signals with K_2_S_2_O_8_ as a coreactant [83]. The MOFs served not only as carriers to enrich ECL luminophores but also as catalysts for oxygen reduction to promote the ECL generation of SO_4_^•−^/O_2_ system, eventually realizing the ultrasensitive detection of the carbohydrate antigen 24-2 with a detection limit of 0.015 mU/mL. Deng et al. developed an ECL immunosensor for the detection of procalcitonin using manganese dioxide nanoflowers and ZnS QDs as dual ECL emitters [84]. In this strategy, abundant Au nanoparticles (AuNPs) and ZnS QDs were incorporated into the nanoflowers to improve the conductivity and ECL efficiency. Meanwhile, manganese dioxide nanoflowers could act as ECL emitters and co-reaction accelerators to further amplify the ECL signal, achieving the sensitive detection of procalcitonin with a detection limit of 0.033 pg/mL.

In contrast to “signal-on” immunosensors, ECL intensity of the “signal-off” immunosensors is negatively correlated with the concentration of targets, which is mainly achieved by quenching ECL signals of QDs in various ways after the formation of sandwich immunocomplexes. Currently, there are mainly two ECL quenching strategies reported in the literature, namely resonance energy transfer (RET) and competitive consumption of coreactants [75,85]. The former usually requires a donor-acceptor pair, in which the ECL emission spectrum of the donor partially overlaps with the ECL absorption spectrum of the acceptor [86]. Since donors and acceptors are fixed on the electrodes and the secondary antibodies, respectively, and far away from each other, the ECL-RET cannot occur in the absence of immunoreactions. After the capture of target antigens, the formation of immunocomplexes narrows the distance between donors and acceptors, meeting the distance requirement for RET. Then ECL emission of the donor fixed on the electrode is significantly quenched, establishing a quantitative relationship between ECL intensity and the concentration of antigens. Typical donor-acceptor pairs include two QDs with different ECL emission wavelength, or QDs and corresponding quenching molecules such as benzoquinone [87,88].

In addition, the “signal-off” immunosensors based on the competitive consumption of coreactants are mainly constructed by labeling electrocatalysts with oxygen reduction catalytic activity on the secondary antibodies, which can selectively catalyze the reduction of dissolved oxygen to produce H_2_O instead of H_2_O_2_, thus reducing the ECL intensity of QDs with H_2_O_2_ as coreactants [75,89]. In this way, the ECL intensity of QDs is negatively correlated with the concentration of antigens, enabling the quantitative detection of trace antigens. As shown in Figure 6d, hemin-functionalized graphene sheet labeled on the secondary antibodies can act as the highly efficient electrocatalyst for the reduction of O_2_ via a four-electron pathway, which greatly prevents the formation of H_2_O_2_ as a QD-based ECL coreactant and then quenches the ECL emissions of QDs, thus achieving the sensitive detection of CEA.

#### 3.1.2. Immunoassay Based on DNA/RNA Aptamers

Aptamers are DNA/RNA oligonucleotide fragments or short peptides with a high affinity for specific targets [90]. Compared with antibodies, aptamers have the advantages of low cost, being easy to synthesize and having good stability, showing the potential to replace antibodies as the emerging target receptors [91]. In recent years, the application of aptamers in QD-based ECL immunoassay has been explored due to its high specificity and selectivity to the target [92,93,94,95]. For example, Zhu et al. developed a QD-based ECL biosensor for the detection of lysozyme through the recognition of lysozyme by aptamers [92]. Due to the competitive binding of complementary DNA labeled by QDs with lysozyme to the aptamer fixed on the electrode, the ECL intensity of QDs is negatively correlated with the concentration of lysozyme, thus enabling the quantitative detection of lysozyme. Later, Jie et al. proposed a multiple DNA cycle amplification strategy for thrombin assay based on ECL quenching of QDs [96]. In this strategy, QDs was first fixed to the electrode, followed by the conjugation to the hairpin DNA labeled with AuNPs on the other end. In this case, the ECL signals of QDs could be efficiently quenched by AuNPs in the absence of thrombin. In the presence of thrombin, the recognition of thrombin by aptamers led to the release of complementary DNA (cDNA), which then hybridized with the loop of hairpin DNA and induced the recognition of this region by endonuclease, resulting in the break of the hairpin DNA and the release of AuNPs. ECL signals were greatly amplified after multiple cycles of the cleavage reaction, thus improving the detection sensitivity of thrombin. In addition, Feng et al. constructed a dual-stimuli responsive ECL biosensor for the detection of pathogenic bacterial [97]. In the absence of bacterial, silver nanocluster-labeled hairpin DNA quenched ECL emission from CdS QDs by resonance energy transfer. In the presence of bacteria, the hairpin DNA was cleaved and silver nanoclusters were released from the surface of CdS QDs, and then probe DNA labeled with AuNPs was introduced to pair with the residual sequence of hairpin DNA, resulting in the enhancement of ECL emission by the surface plasmon resonance effect.

In addition, target-triggered ratiometric sensing strategies have also been used to construct aptamer-based ECL immunosensors. For example, Han et al. prepared a novel MOFs/Au/G-quadruplex as both quencher and enhancer to fabricate a target-triggered ratiometric ECL sensor for accurate detection of prostatic specific antigen (PSA) [98]. In this work, CdSe/ZnS QDs and luminol were used as dual-potential-dependent ECL emitters for ratiometric sensing. After the sequential hybridization of cDNA labelled with QDs, PSA aptamer and probe DNA linked with Au/hemin@MOFs-DNAzyme and the further competition of PSA, the probe DNA would keep away from the electrode, causing a switchover of ECL signals of QDs-luminol pairs from an “off-on” state to an “on-off” one. Therefore, PSA was sensitively quantified by an ECL intensity ratio of two emitters. 

#### 3.1.3. Multiplex Immunoassay

The ECL immunosensor discussed above can only achieve the detection of a single antigen in a single run. In fact, the demand for simultaneous detection of multiple targets in immunodiagnostics has increased rapidly, which puts forward higher requirements for the signal-resolving strategies of multiple ECL emitters [4]. According to the difference in signal-resolving strategies, the multiplex immunosensors based on the ECL of QDs can be divided into potential-resolved and wavelength-resolved formats, both of which depend on the adjustment of the band gap by tuning the size and composition of QDs [99,100,101,102].

**Figure 7:**
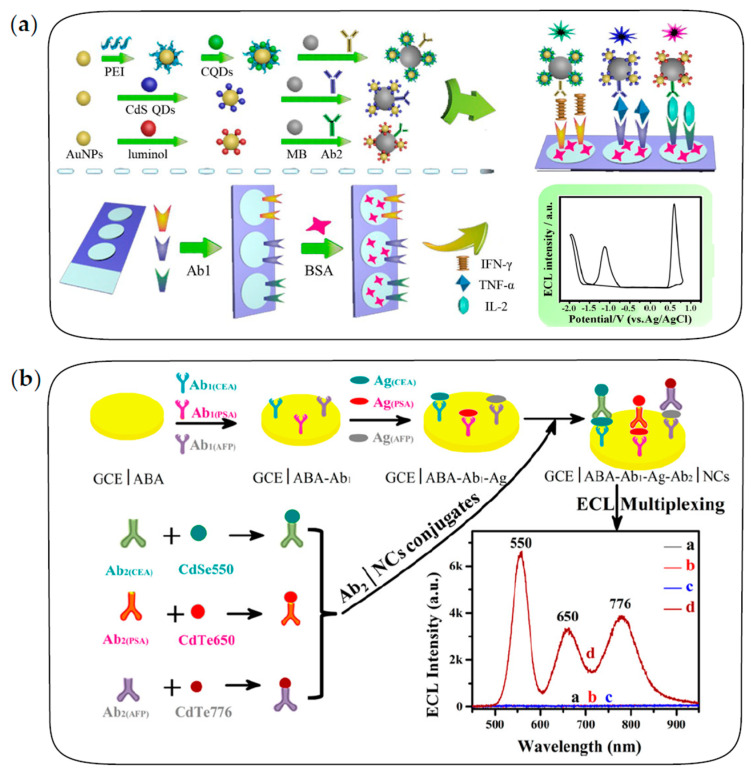
The fabrication procedures of multiplexed ECL immunosensors. (**a**) The multiplexed ECL immunosensor based on potential-resolved strategies for simultaneous detection of three LTBI markers. Reprinted with permission from ref. [100]. Copyright 2017 American Chemical Society. (**b**) The multiplexed ECL immunosensor based on wavelength-resolved strategies for simultaneous detection of three tumor markers. Reprinted with permission from ref. [26]. Copyright 2018 American Chemical Society.

Yang et al. first constructed ECL immunosensors based on potential-resolved strategies for simultaneous determination of triple latent tuberculosis infection (LTBI) markers [100]. As shown in Figure 7a, three antibodies are separately immobilized on three spatially-resolved areas of a patterned ITO electrode to capture the corresponding LTBI markers; luminol, carbon QDs and CdS QDs are integrated onto AuNPs and magnetic beads sequentially to fabricate potential-resolved ECL nanoprobes with signal amplification. In the presence of LTBI markers, the formation of the immunocomplex generates three potential-resolved ECL signals during one potential scanning and the ECL intensities reflect the concentrations of three LTBI markers, respectively. Recently, Zeng et al. constructed a dual-signal ECL immunosensor based on sandwich structure and a magnetic separation technique for the simultaneous detection of carbohydrate antigen 125 (CA125) and human epithelial protein 4 (HE4) markers of ovarian cancer [103]. Eu MOFs loaded with isoluminol and AuNPs generated a strong anodic ECL signal, and the composite of CdS QDs and a Cu single-atom catalyst could act as a cathodic ECL emitter and catalyze the reduction of H_2_O_2_ to produce a large amount of ^•^OH and O_2_^•−^, therefore achieving remarkable bipolar ECL signals. With two potential-resolved ECL luminophores as labels, this platform successfully performed simultaneous detection of ovarian cancer markers with detection limits of 0.37 pg/mL and 1.58 pg/mL for CA125 and HE4, respectively.

Guo et al. first proposed the ECL-immunosensing strategy with multicolor QDs as labels for the simultaneous determination of two different tumor markers, AFP and CEA [99]. Subsequently, Zou’s group has conducted several explorations on wavelength-resolved ECL immunosensors with QDs as labels [26,101]. For example, Zou and co-workers proposed a spectrum-resolved triplex-color ECL multiplexing immunoassay for the simultaneous determination of three different tumor markers, CEA, PSA and AFP [26]. As shown in Figure 7b, three QD labels with different emission wavelengths are conjugated with three detecting antibodies of CEA, PSA and AFP, respectively. In the presence of targets, three immune sandwich structures are formed simultaneously, and then the maximum ECL intensity of each label can be used to quantify the corresponding antigen. It is observed that ECL emissions of three QDs are clearly distinguished in the spectral range of 500–900 nm to quantify the concentration of three antigens in a single run.

It can be seen that the construction of multiplex ECL immunosensors significantly depends on the development of QD synthesis strategies. QDs with narrow emission spectra and continuously tunable wavelength are more favorable for ECL multiplex immunoassay. Therefore, it is still of great importance to develop high-quality synthesis strategies for QDs in aqueous systems.

### 3.2. Nucleic Acid Analysis

#### 3.2.1. Nucleic Acid Analysis Based on Sandwich Structures

In recent years, QD-based ECL biosensors with sandwich structures have been widely explored in nucleic acid analysis. QD-based DNA/RNA biosensors with sandwich structures generally consist of a capture probe, a target and a detection probe. Capture probes are attached to the solid substrates, which can be electrodes or magnetic beads, for the specific recognition of targets [104,105]; QDs as ECL emitters are conjugated to detection probes or electrodes [106,107].

**Figure 8:**
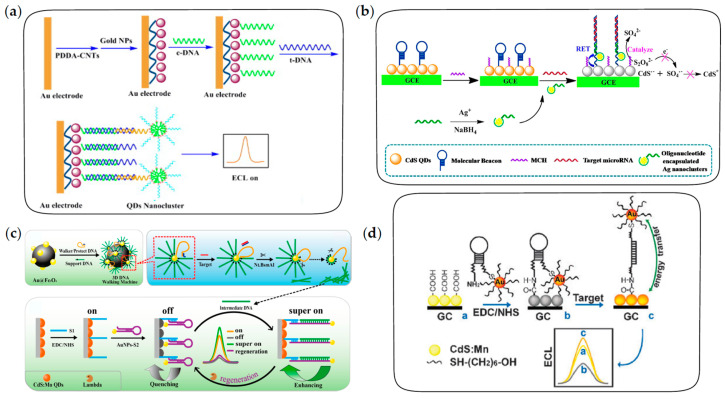
The fabrication procedures of ECL biosensors for nucleic acid analysis. (**a**) The “signal-on” ECL biosensor with sandwich structure for DNA detection. Reprinted with permission from ref. [108]. Copyright 2014 Elsevier. (**b**) The “signal-off” ECL biosensor with sandwich structure based on target-induced ECL quenching for microRNA detection. Reprinted with permission from ref. [107]. Copyright 2015 American Chemical Society. (**c**) The ultrasensitive ECL biosensor based on 3D DNA walking machine and localized surface plasmon resonance (LSPR) enhancement strategy for microRNA detection. Reprinted with permission from ref. [109]. Copyright 2017 American Chemical Society. (**d**) The distance-dependent ECL biosensor based on RET and LSPR effects for DNA detection. Reprinted with permission from ref. [63]. Copyright 2009 Royal Society of Chemistry.

The “signal-on” ECL biosensors for nucleic acid analysis are constructed by labeling QDs on the detection probes, in which ECL intensity is positively correlated with the concentration of targets. For example, Jie et al. constructed a “signal-on” ECL biosensor with dendritic QDs nanocluster as emitters for the detection of target DNA [108]. The fabrication of biosensors is depicted in Figure 8a. After the sandwich structure is formed through the sequential recognition of targets by capture probes and detection probes, dendritic nanoclusters enriched with a large number of QDs are captured on the electrode to generate an amplified ECL signal for the sensitive detection of target DNA. Unlike the above process, the “signal-off” ECL biosensors are constructed by the binding of QDs on the electrodes and subsequent target-induced ECL quenching. Xu et al. developed a novel ECL biosensor based on the quenching effect of Ag nanoclusters on CdS QDs for the sensitive detection of microRNA [107]. As shown in Figure 8b, molecular beacons are immobilized on GCE modified with CdS QDs through the formation of a Cd-S bond, followed by adding 6-mercapto-1-hexanol (MCH) to block non-specific sites. After being hybridized with the target microRNA and oligonucleotide-encapsulated Ag nanoclusters sequentially, the hairpin structure opens up and then Ag nanoclusters are in close proximity to CdS QDs on the modified GCE. Ag nanoclusters can not only quench the ECL emission of CdS QDs by RET but also catalyze the electrochemical reduction of K_2_S_2_O_8_ to promote the consumption of coreactants. Based on the dual quenching effects, a sensitive ECL biosensing of microRNA is achieved with a wide linear range and acceptable selectivity.

In addition, various signal amplification strategies have been widely explored to further improve the sensitivity of nucleic acid analysis, such as target-induced recycling amplification, cascade amplification strategies and strand displacement reactions [110,111,112,113,114,115,116]. For example, Yuan et al. constructed a novel ECL biosensor to achieve the ultrasensitive detection of microRNA by combining target recycling amplification and double-output conversion strategies [110]. In this case, the ECL efficiency of QDs was improved by conjugation with ruthenium complexes, in which ECL-RET took place efficiently because of the short path of energy transfer. Target-induced DNA polymerization and the subsequent release of synthesized reporter DNA enabled a small number of microRNA to be successfully transferred into a large number of reporter DNA strands, which could capture numerous QD-labeled signal probes on the sensing surface to realize the sensitive ECL response to target microRNA. Song et al. developed the “signal-on” ECL biosensor for the detection of microRNA-141 based on a dual isothermal enzyme-free strand displacement reaction [114]. In the presence of trace target microRNA, the strand displacement reaction was triggered and abundant mimic targets were released, thus achieving amplification of the target. In the detection process, capture probes were fixed on a AgInZnS QD-modified electrode and paired with ferrocene-labeled probes, causing the ECL signal to be in an “off” state. However, the competitive binding of mimic targets and ferrocene-labeled probes to capture probes led to the release of ferrocene from the electrode and the recovery of ECL signals, thus enabling the sensitive biosensing of microRNA-141 with a low detection limit of 33.3 aM.

In recent years, the DNA walking machine has aroused increasing interest for its efficient self-assembly and signal amplification abilities, and it has also been used in QD-based DNA/RNA biosensing [117,118]. Researchers have made some explorations of QD-based ECL biosensors amplified by a DNA walking machine for ultrasensitive detection of microRNA, including dual-legged DNA walkers and a three-dimensional (3D) DNA walking machine [109,119,120]. As shown in Figure 8c, an ultrasensitive ECL biosensor for microRNA detection is constructed based on a 3D DNA walking machine and localized surface plasmon resonance (LSPR) enhancement strategy. In this strategy, Au@Fe_3_O_4_ is used to immobilize supporting DNA and walking DNA paired with protecting DNA to form the 3D DNA walking machine. Once paired with protecting DNA, walking DNA is locked and cannot work. In the presence of target microRNA, walking DNA is released by the competitive pairing of target microRNA and protecting DNA, followed by pairing with supporting DNA to form the recognition site to be released again under the shearing of a Nt.BsmAl nicking endonuclease. Subsequently, the released walking DNA moves along the surface of Au@Fe_3_O_4_ and repeats the process, generating a large number of intermediate DNA strands in the presence of trace target microRNA. Finally, intermediate DNA is used to open the hairpin structure on the electrode, generating LSPR enhancement effect on ECL intensity of QDs. Dual signal amplification strategies significantly improve the sensitivity of microRNA detection. Therefore, the 3D DNA walking machine has higher efficiency of payload release and superior signal amplification than those of the traditional DNA walking machines, allowing ultrasensitive detection of microRNA.

#### 3.2.2. Nucleic Acid Analysis Based on Recognition of Complementary Sequences

Another type of QD-based ECL biosensor for nucleic acid analysis is constructed through the specific recognition of the target by hairpin DNA. In this strategy, the film of QDs with stable ECL emission is generally coated on the electrodes, followed by target-induced ECL enhancement or quenching to achieve a sensitive response of the ECL intensity to the target. Currently, ECL quenching strategies reported in the literature include RET effects and competitive consumption of coreactants, while ECL enhancement strategies are mainly based on the LSPR effect [121,122,123,124]. Among them, the RET quenching effect and LSPR enhancement effect are both derived from the interaction between noble metal nanocrystals and ECL emitters. The dominant effect mainly depends on the distance between the noble metal nanocrystals and ECL emitters. Generally, the RET effect dominates at short range, while the LSPR effect dominates at long range. For instance, Xu et al. first proposed a distance-dependent ECL quenching or enhancement strategy based on the interaction between AuNPs and CdS:Mn QDs for DNA detection [63]. As shown in Figure 8d, hairpin DNA with AuNPs labeled in the terminal was covalently attached to GCE modified with CdS:Mn QDs by amidation. Upon the occurrence of the hybridization with target DNA, ECL quenching arising from the RET effect at short range was converted to ECL enhancement arising from the LSPR effect at long range, the combination of which provided a high sensitivity for DNA detection.

In addition, ECL quenching strategies based on competitive consumption of coreactants are also used to construct biosensors for nucleic acid detection. Ju et al. developed a label-free QD-based ECL system for DNA assay based on the consumption of coreactants by the electrocatalytic reduction of dissolved oxygen with DNAzyme [123]. In this system, the label-free hairpin DNA was attached to the electrode modified with QDs. Upon the hybridization of the hairpin with target DNA in the presence of hemin, the specific sequence conjugated with hemin to form a G-quadruplex architecture, which showed a high catalytic activity for electrochemical reduction of dissolved oxygen, leading to a decrease in the ECL signal.

By introducing QDs with high ECL efficiency into the construction of nucleic acid biosensors, the sensitivity of DNA/RNA detection has been significantly improved, but there is still a lack of studies on high-throughput DNA/RNA detection. More efforts need to be devoted to develop QD-based nucleic acid biosensing strategies with both high sensitivity and high throughput.

### 3.3. Small Molecules and Ions Detection

#### 3.3.1. Target-Induced ECL Quenching

Since many molecules and ions have quenching effects on ECL emission of QDs, target-induced ECL quenching has been the most classical sensing strategy for the detection of small molecules and ions. The quenching mechanisms of ECL include quenching of excited states of QDs [125,126,127,128], quenching of coreactant radicals [64,129,130,131], inhibition of the electrochemical process [132,133,134,135], competitive consumption of coreactants [136] and destruction of QD structures [137,138]. Sensing strategies for small molecules and ions based on the various ECL quenching mechanisms are described in this section.

Dopamine and benzoquinone are common model analytes that can quench QD*. For example, Ag_2_Se QDs with near-infrared ECL emission were used to construct the sensor for dopamine based on its quenching effect on the excited state [125]. The energy level of dopamine was between the valence band and conduction band of Ag_2_Se QDs, so the quenching effect on the near-infrared ECL emission of QDs resulted from the process of electron transfer. In addition, the energy transfer between excited ZnSe QDs and benzoquinone produced by the oxidation of hydroquinone was used for the bio-detection of hydroquinone [127]. As an efficient coreactant promoting ECL emission of ZnSe QDs, K_2_S_2_O_8_ with strong oxidizing properties could oxidize hydroquinone to benzoquinone, which thus displayed a strong inhibition on ECL emission.

**Figure 9:**
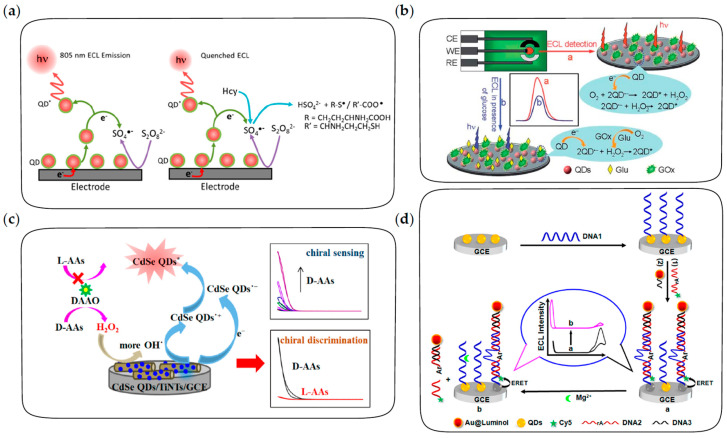
The fabrication procedures of ECL biosensors for the detection of small molecules and ions. (**a**) ECL biosensor based on quenching of coreactant radicals for the detection of homocysteine in blood. Reprinted with permission from ref. [130]. Copyright 2018 American Chemical Society. (**b**) ECL biosensor based on the competitive consumption of coreactants for the detection of glucose. Reprinted with permission from ref. [139]. Copyright 2012 Royal Society of Chemistry. (**c**) ECL biosensor based on selective production of coreactants for the discrimination and quantification of the isomers of chiral amino acids. Reprinted with permission from ref. [140]. Copyright 2022 American Chemical Society. (**d**) DNA-aptamer based ratiometric ECL biosensor using QDs and luminol as dual emitters for the detection of Mg^2+^. Reprinted with permission from ref. [141]. Copyright 2014 American Chemical Society.

Thiol compounds and ascorbic acid have a strong quenching effect on coreactant radicals by the electron-transfer process and thus inhibit ECL emission of QDs, which has been used to construct ECL biosensors for these molecules. For example, based on the quenching effect of ascorbic acid on SO_4_^•−^, the activity of alkaline phosphatase was determined indirectly according to the concentration of ascorbic acid, which was generated in the hydrolysis reaction of L-ascorbic acid 2-phosphate sesquimagnesium catalyzed by alkaline phosphatase [129]. In addition, homocysteine was a potent radical quencher for near-infrared ECL of CdSeTe/ZnS QDs [130]. As shown in Figure 9a, quenching of SO_4_^•−^ can occur via the sulfydryl terminal or carboxyl terminal of homocysteine, then thiol radicals or carboxylate radicals are formed and dimerized rapidly, establishing a quantitative relationship between the ECL intensity of QDs and the concentration of homocysteine in the blood. In addition, *p*-nitrophenol is also an effective quencher. Mao et al. developed an ECL biosensor for the detection of *p*-nitrophenol based on its quenching effect on ECL signals [131]. A large number of CdSe QDs were loaded into the pores of Zr-based porphyrin MOFs to amplify ECL signals, and the effective quenching effect of *p*-nitrophenol on ECL led to a detection limit as low as 0.03 ppb.

The quenching effect of nitrites on the ECL emission of QDs followed an “electrochemical oxidation inhibition” process [132]. The presence of nitrites produced a large voltage drop and made the practical potential less than the applied potential, leading to a weak ECL emission. A hydroquinone/horseradish peroxidase (HRP)/H_2_O_2_ system was used as a model system to construct an ECL biosensor for the detection of hydroquinone [136]. HRP catalyzed the enzymatic reaction of hydroquinone and H_2_O_2_, leading to the consumption of coreactants and thus the quenching of ECL. In addition, ECL sensors for metal ions with stronger metal-S interaction than a Cd-S bond were constructed based on the structure destruction of CdSe QDs [137]. The competitive binding of Cu^2+^ to the stabilizer led to the precipitation of QDs and thus the quenching of ECL emission, establishing a negative correlation between the ECL intensity of QDs and the concentration of Cu^2+^. This strategy could be extended to the rapid detection of other cations with strong metal-S interactions [138].

#### 3.3.2. Coreactant Concentration-Dependent Biosensing Strategy

In this strategy, the concentration of target molecules is correlated with the concentration of coreactants and thus with the ECL intensity of QDs, which can be achieved either by considering target molecules as coreactants or by the target-induced consumption or production of coreactants. For example, DBAE and TPrA were sensitively detected as ECL coreactants of CdTe and ZnSe QDs, respectively [53,142]. In addition, an ECL biosensor for the detection of glucose was constructed based on the competitive consumption of dissolved oxygen, which acted as a coreactant in the QD-based ECL process [139]. As shown in Figure 9b, under the catalysis of glucose oxidase, the oxidation of glucose leads to the consumption of a large amount of dissolved oxygen and the production of H_2_O_2_. Due to the higher efficiency of dissolved oxygen as a coreactant than H_2_O_2_, the ECL intensity of QDs decreases in the presence of glucose, resulting in a sensitive response of the ECL biosensor to glucose. Alternatively, AuNPs are efficient glucose oxidase-mimics to catalyze the oxidation of glucose, which can also be used to construct ECL biosensors for the detection of glucose based on the competitive consumption of dissolved oxygen [143]. Moreover, as shown in Figure 9c, due to the selectivity of D-amino acids oxidase on the oxidation of D-amino acids rather than L-amino acids, the isomers of chiral amino acids can be effectively discriminated and quantified based on the ECL response of CdSe QDs [140].

#### 3.3.3. DNA Aptamer-Based Biosensing Strategy

DNA aptamers are efficient probes for the specific recognition of some molecules and metal ions, which have also been extensively explored for ECL biosensing of these analytes [141,144,145,146]. The aptamer-based ECL biosensor for adenosine 5′-triphosphate (ATP) detection was developed based on the aptamer-ATP specific affinity and the rule of Watson–Crick base pairing [144]. After the formation of aptamer-ATP complexes on the electrode, cDNA was hybridized with the remaining free probes. Subsequently, QDs were labeled through the biotin-avidin reaction in the existence of biotin-modified cDNA. Therefore, the ECL intensity of QDs showed sensitive response to ATP. In addition, DNA aptamers with a hairpin structure were also used to construct an ECL biosensor for the detection of Pb^2+^ [145]. In the presence of Pb^2+^, the “stem-loop” structure of hairpin aptamer opened up, followed by the formation of a G-quadruplex and the conjugation of QDs to the terminal amino, thus ECL emission was significantly enhanced by the addition of Pb^2+^. In addition, the DNAzyme-triggered ECL ratiometric biosensing strategy was developed for the sensitive detection of Mg^2+^ using CdS QDs and luminol as dual-potential ECL emitters [141]. As depicted in Figure 9d, the biosensor consists of DNAzyme strands labeled with CdS QDs as capture probes and cathodic ECL emitters, luminol-reduced AuNPs as anodic ECL emitters and Mg^2+^ substrate strands labeled with cyanine dye (Cy5) fluorophores as quenchers. In the absence of Mg^2+^, luminol shows intense anodic ECL emission while the cathodic ECL of QDs is quenched by energy transfer with Cy5. In the presence of Mg^2+^, DNAzyme cleaves the substrate strand, followed by the release of Cy5 and luminol from the electrode, resulting in the recovery of cathodic ECL from QDs and the decrease in anodic ECL from luminol simultaneously. Therefore, the DNAzyme-triggered ratiometric ECL strategy provides a reliable and sensitive method in biosensing.

## 4. Conclusions and Perspectives

In conclusion, as promising ECL luminophores with excellent optical properties, semiconductor QDs with various structures and compositions have been widely explored in both luminescent mechanism and biosensing applications. In this review, we give a comprehensive summary of recent advances in using semiconductor QDs as ECL luminophores. At first, the relationship between the structure of QDs and ECL performance is discussed in detail. ECL has been shown to be more sensitive to surface defects than PL, which can quench the band-edge emission, thus leading to a decrease in ECL efficiency. The ECL efficiency of QDs can be significantly improved by eliminating these defects and introducing a variety of ECL-enhancing strategies. Subsequently, the ECL mechanism of semiconductor QDs with various coreactants is introduced, mainly including annihilation and coreactant-containing ECL. On the basis of understanding the ECL mechanism of QDs, the application of semiconductor QDs in ECL biosensing is summarized comprehensively. With the in-depth exploration over the past two decades, semiconductor QD-based ECL techniques have been widely applied in immunoassay, nucleic acid analysis, detection of small molecules and other fields, which has greatly improved the detection performance of ECL biosensors (the detection limit is as low as ~aM) and broadened their application fields.

However, due to the complexity of the structure of QDs, there are still some problems to be solved, which puts forward higher requirements for future studies on QD-based ECL. Firstly, as discussed above, various surface passivation strategies such as core-shell structure construction and surface ligand passivation have been proposed to improve the optical properties of QDs. However, inorganic shell and hydrocarbon ligands acting as insulating layers can significantly hinder the process of charge transfer and decrease the ECL efficiency of QDs. The effects of inorganic shell and surface ligands on ECL properties of QDs should be studied systematically. Secondly, due to the larger size of QDs than conventional organic dyes, it is difficult to label QDs on corresponding active sites and reduce non-specific adsorption in biological samples; thus, the application of QDs in ECL imaging is still less studied. Finally, QDs are promising luminophores to achieve single-molecule ECL detection for their high luminescence efficiency, which will be one of the hot directions in future research.

## Figures and Tables

**Table 1:** Typical oxidative-reductive coreactants, corresponding ECL reaction steps and conditions.

Coreactants	ECL Reactions	QDs	ECL Potential/V	ECL Conditions	Ref.
C_2_O_4_^2−^	$\mathrm{QD}-e^{-}\to {\mathrm{QD}}^{+•}$	Si	+2.5 V (vs. Ag QRE)	THAP/MeCN	[18]
	$C_{2}O_{4}-e^{-}\to {\mathrm{CO}}_{2}{}^{-•}+{\mathrm{CO}}_{2}$				
	${\mathrm{QD}}^{+•}+{\mathrm{CO}}_{2}{}^{-•}\to {\mathrm{QD}}^{*}+{\mathrm{CO}}_{2}$				
TPrA	$\mathrm{QD}-e^{-}\to {\mathrm{QD}}^{+•}$	CdTe/CdS	+0.5 V (vs. Ag/AgCl)	Tris (pH 8.0)	[54]
	$\mathrm{TPrA}-e^{-}\to {\mathrm{TPrA}}^{+•}\to {\mathrm{TPrA}}^{•}+H^{+}$ ${\mathrm{QD}}^{+•}+{\mathrm{TPrA}}^{•}\to {\mathrm{QD}}^{*}+\mathrm{TPrA}\;\mathrm{fragments}$				
SO_3_^2−^	$\mathrm{QD}-e^{-}\to {\mathrm{QD}}^{+•}$	CdTe	+0.89 V (vs. Ag/AgCl)	Air-saturated PBS(pH 7.5)	[57]
	${\mathrm{SO}}_{3}{}^{2-}-e^{-}\to {\mathrm{SO}}_{3}{}^{-•}$				
	$2{\mathrm{OH}}^{-}+{\mathrm{SO}}_{3}{}^{-•}+2O_{2}\to O_{2}{}^{-•}+{\mathrm{SO}}_{4}{}^{2-}+H_{2}O$				
	$\mathrm{QD}+O_{2}{}^{-•}\to {\mathrm{QD}}^{-•}+O_{2}$ ${\mathrm{QD}}^{-•}+{\mathrm{QD}}^{+•}\to {\mathrm{QD}}^{*}+\mathrm{QD}$				
ITO	${\mathrm{In}/\mathrm{SnO}}_{x}+\mathrm{QD}\to {\mathrm{In}/\mathrm{SnO}}_{x}{}^{-•}+{\mathrm{QD}}^{+•}$	CdTe	+1.17 V (vs. Ag/AgCl)	Air-saturated PBS (pH 7.4)	[58]
	${\mathrm{In}/\mathrm{SnO}}_{x}{}^{-•}+O_{2}\to {\mathrm{In}/\mathrm{SnO}}_{x}+O_{2}{}^{-•}$ $\mathrm{QD}+O_{2}{}^{-•}\to {\mathrm{QD}}^{-•}+O_{2}$ ${\mathrm{QD}}^{-•}+{\mathrm{QD}}^{+•}\to {\mathrm{QD}}^{*}+\mathrm{QD}$				

* THAP, tetrahexylammonium perchlorate; Ag QRE, Ag wire quasi-reference electrode.

**Table 2:** Typical reductive-oxidative coreactants, corresponding ECL reaction steps and conditions.

Coreactants	ECL Reactions	QDs	ECL Potential/V	ECL Conditions	Ref.
CH_2_Cl_2_	$\mathrm{QD}+e^{-}\to {\mathrm{QD}}^{-•}$	CdTe	−1.85 V (vs. SCE)	TBAPF_6_/CH_2_Cl_2_	[21]
	${\mathrm{CH}}_{2}{\mathrm{Cl}}_{2}+e^{-}\to {\mathrm{CH}}_{2}{\mathrm{Cl}}_{2}{}^{-•}\to {\mathrm{CH}}_{2}{\mathrm{Cl}}^{•}+{\mathrm{Cl}}^{-}$				
	${\mathrm{QD}}^{-•}+{\mathrm{CH}}_{2}{\mathrm{Cl}}^{•}\to {\mathrm{QD}}^{*}+{\mathrm{CH}}_{2}{\mathrm{Cl}}^{-}$				
S_2_O_8_^2−^	$\mathrm{QD}+e^{-}\to {\mathrm{QD}}^{-•}$	CdSe	−0.2 V (vs. SHE)	KOH	[38]
	$S_{2}O_{8}{}^{2-}+e^{-}\to {\mathrm{SO}}_{4}{}^{-•}+{\mathrm{SO}}_{4}{}^{2-}$ ${\mathrm{QD}}^{-•}+{\mathrm{SO}}_{4}{}^{-•}\to {\mathrm{QD}}^{*}+{\mathrm{SO}}_{4}{}^{2-}$				
H_2_O_2_	$\mathrm{QD}+e^{-}\to {\mathrm{QD}}^{-•}$ $H_{2}O_{2}+e^{-}\to {\mathrm{HO}}^{•}+{\mathrm{HO}}^{-}$ ${\mathrm{QD}}^{-•}+{\mathrm{HO}}^{•}\to {\mathrm{QD}}^{*}+{\mathrm{HO}}^{-}$	CdSe/ZnS	−0.85 V (vs. Ag/AgCl)	PBS (pH 7.4)	[61]
O_2_	$\mathrm{QD}+e^{-}\to {\mathrm{QD}}^{-•}$	CdSe	−1.1 V (vs. Ag/AgCl)	KNO_3_/ Air-saturated PBS (pH 9.3)	[39]
	$O_{2}+H_{2}O+2e^{-}\to {\mathrm{HOO}}^{-•}+{\mathrm{HO}}^{-}$ ${2\mathrm{QD}}^{-•}+{\mathrm{HOO}}^{-•}+H_{2}O\to {2\mathrm{QD}}^{*}+3{\mathrm{HO}}^{-}$				

* TBAPF_6_, tetra-*n*-butylammonium hexafluorophosphate; SCE, standard calomel electrode; SHE, standard hydrogen electrode.

## Data Availability

Not applicable.
